# Teachers’ and students’ perspectives on the needs of community practice teachers: a cross-sectional study

**DOI:** 10.1186/s12909-023-04456-1

**Published:** 2023-06-30

**Authors:** Lin Tao, Ying Yang, Xiaolin Ma, Lan Fu, Suzhen Liu

**Affiliations:** 1 Cancer Day-Care Unit, Department of Medical Oncology, Cancer Center, West China Hospital, Sichuan University/ West China School of Nursing, Sichuan University, 610041 Chengdu, China; 2 Department of Nursing, West China Hospital, Sichuan University/ West China School of Nursing, Sichuan University , 37 Guoxue Road, WuHou District, 610041 Chengdu, China

**Keywords:** Community practice teacher, Teaching needs, Practice teaching, Teaching behavior

## Abstract

**Background:**

An accurate assessment of teaching needs is necessary to ensure targeted teacher training programs are developed and implemented to improve teaching outcomes. The assessment of teaching needs from different perspectives helps to identify teaching needs more accurately. Therefore, based on the different perspectives of teachers and students, this study aimed to identify and evaluate the needs of community practice teachers by measuring discrepancies between perceived teaching importance and actual teaching performance, with a focus on the influencing factors.

**Methods:**

A survey was circulated to 220 teachers in 36 community health service centers and 695 students in 6 medical schools in Southwest China. The participants anonymously completed the teacher or student version of the Chinese version of the Teacher Teaching Needs Questionnaire, which is predominantly used to assess the teaching needs of teachers. Both versions of the questionnaire include 27 items, covering 3 dimensions (including teaching skills, teaching environments, and teaching contents). The ordinal logistic regression was conducted to explore the factors that influenced teaching needs.

**Results:**

The teachers and students produced overall self-evaluated teaching needs scores of 0.61 and 0.62 respectively. The teachers from provincial capital cities and low-educated teachers had teaching needs that are lower (OR = 0.641,95% CI: 0.461–0.902, OR = 15.352, 95% CI: 1.253–26.815, separately). Teachers with < 3 years of teaching experience had higher teaching needs (OR = 3.280, 95% CI: 1.153–10.075) than those with > 10 years of experience. Compared with teachers who self-evaluated their teaching outcomes as poor, those who reported extremely excellent (OR = 0.362, 95% CI: 0.220–0.701), excellent (OR = 0.638, 95% CI: 0.426–1.102), and ordinary (OR = 0.714, 95% CI: 0.508–1.034) teaching outcomes had lower teaching needs. Compared with teachers who self-evaluated their teaching abilities as poor, those who reported extremely excellent (OR = 0.536, 95% CI: 0.313–0.934), excellent (OR = 0.805, 95% CI: 0.518–1.269), and ordinary (OR = 0.737, 95% CI: 0.413–1.322) teaching abilities had lower teaching needs.

**Conclusions:**

Greater assistance should be provided to teachers with lower levels of education, fewer than three years of teaching experience, and who are located in non-capital cities, as these individuals require additional efforts to strengthen competencies. The education department should pay more attention to teacher feedback on practical outcomes and teaching abilities, as this can be used to devise the best teacher development plans.

**Trial registration:**

Not applicable.

## Background

As “gatekeepers” of health for local populations, community health nurses provide a series of critical services, including preventive care, common disease referrals, rehabilitation, chronic disease management, and palliative care [1, 2]. At the same time, they are tasked with providing practical education to nursing students. Given this dual professional role, it is equally important for them to have adequate clinical skills and teaching competencies. In this context, community practice teachers serve as health nurses who work at community health service centers and undertake practical teaching tasks, thus forming an essential link between academic programs and clinical practice [3–5]. In China, the relevant administrative departments do not prioritize the development of community health service centers, and the management of these institutions is not clear [6]. Until 2010, the construction of urban community health service system has been basically completed. Inadequate attention, insufficient funds and a lack of health professionals have led to a late start and lagging development in community health service centers. However, the Ministry of Education has only recently designated community health service centers as practical bases for nursing undergraduates [7, 8]. In light of this, the training of community nurses should be taken seriously. To date there is no normative documentation related to the training of community nurses’ teaching ability in China. It is of vital importance to develop a new training program that focusses on the training of teachers’ teaching competence.

When formulating teacher training plans, relevant departments have often solicited the opinions of experienced teachers, asking them what skills or content should be listed as training items [9, 10]. These teachers usually identify the most important skills or content in teaching activities as the primary training focus These include teaching methods and techniques [11, 12]. However, teaching skills or content that are important in teaching activities are not necessarily areas wherein teachers struggle. According to Professor Quirk [13], only those teaching areas that are both important and poorly executed should be listed in training programs. The concept of “teaching need” was defined as the gap between perceived teaching importance and actual teaching performance [14]. If teachers understand this gap, they can improve their teaching abilities through continuous learning [15]. However, those who do not recognize this gap may be satisfied with existing conditions and will therefore remain at risk of incompetence in diverse teaching tasks. Perceived teaching importance and actual teaching performance are two elements of teaching need [13]. The former refers to perceptions of which behaviors are considered important, while the latter refers to current or actual performance. Real teaching needs can indirectly be evaluated by clarifying the gap between these elements. Compared to simple direct questions (e.g., “what do you need?”), the indirect evaluation method can reveal what is “desired” relatively objectively [16, 17]. Moreover, teachers can use this method independently, or it can be employed indirectly to solicit the perspectives of others (e.g., peers, superiors, or educated persons such as students, patients, and residents). As the main participants in teaching activities, students’ perspectives significant, while not always consistent with those of teachers [4, 18–20]. According to Javed [21], incongruousness existed between the students and teachers, about the quality of clinical feedback provided to the students. As such, this study assessed teaching needs based on the perspectives of teachers and their students, thus providing an objective and scientific basis for the targeted development of community practice teacher training.

## Methods

### Sampling procedures and participants

This study adopted a multicenter, cross-sectional, descriptive survey approach. This study was performed from January 2020 to February of 2021. Study participants were fourth-year undergraduates at 6 medical schools and teachers working in 36 community health service centers. We used a phased sampling method to select samples as follows: Step 1: We contacted members of the community nursing special committee of the Sichuan Nursing Society in 21 cities (prefectures) and obtained a list of community health service centers that have undertaken community practical teaching tasks. These institutions are mainly located in the provincial capital city of Chengdu and 11 prefecture-level cities (Deyang, Mianyang, Guangyuan, Dazhou, Nanchong, Guang’an, Suining, Luzhou, Leshan, Ya’an, and Panzhihua). Step 2: Using a convenience sampling method, community nursing committee members from 11 prefecture-level cities contacted 36 community health service centers that were willing to cooperate in completing this survey and had undertaken community practical teaching tasks. Step 3: Using a cluster sampling method, all community practice teachers from 36 community health service centers were included as research subjects. Step 4: Using the cluster sampling method, all undergraduate nursing students from six medical colleges who completed the survey in the 36 community health service centers during the survey period were included as the research subjects. For teachers, the following inclusion criteria were set: (1) formally employed and (2) undertaken community practice teaching tasks. However, we excluded those who were absent during the survey period (e.g., due to extended sick leave or maternity leave). For students, the following inclusion criteria were set: (1) undergraduate status, (2) completed practical learning tasks, and (3) the duration of the internship is 2–4 weeks. The online survey was conducted at the end of a community internship. They are strongly encouraged to complete the questionnaire, but their participation remains voluntary. The names and other personal information of the study participants were protected. The clinical trial and biomedical ethics committee of West China Hospital of Sichuan University approved the study in accordance with the Helsinki Declaration. Teachers and students were informed about the study and signed consent form. The main outcome measures of this study were the importance of teaching needs and performance evaluation.

The sample size was estimated from the mean of the sample, referring to the results of previous studies: For teachers, the mean and standard deviation of importance were 4.28 and 0.43, respectively, and the mean and standard deviation of performance were 3.67 and 0.65, respectively. Set α to 0.05 and error to 0.1. The sample size calculated by Pass15.0 software was 148 and 165, respectively, and the data missing rate is 20%. The final sample size is 185 and 207, respectively. We chose the largest sample size and this meant that we needed to have a sample population of at least 207 teachers. For students, the mean and standard deviation of importance were 4.20 and 0.64, respectively, and the mean and standard deviation for performance were 3.56 and 0.68, respectively. Set α to 0.05 and error to 0.1. The sample size calculated by Pass15.0 software was 148 and 165, respectively, and the data missing rate is 20%. The final sample size is 185 and 207, respectively. The sample size of the students was based on the teacher’s sample size. We used a cluster sampling method to include all the students who met the criteria during the survey period. In the end, 695 students completed the survey.

### Instruments

The participants completed two-part questionnaires. The first part asked for general information, including basic demographics and teaching data. For teachers, this included community setting, gender, age, education, profession, teaching time, self-evaluation of teaching ability, and self-evaluation of training needs. For students, this included sex and age.

The second part contained a teaching needs assessment scale, with different versions for teachers and students. These sections were revised based on the teaching needs questionnaire designed by Quirk et al. (2002) [15]. Tao et al. [11, 22] translated it into Chinese and revised the student version of the teacher needs assessment scale based on the teacher version. The teachers’ version [11] and students’ version [22] have excellent psychometric properties. Specifically, the scale consisted of three dimensions across 27 items on practice teaching behaviors, including teaching skills (10 items), teaching environments (6 items), and teaching contents (11 items), all of which were answered on a 5-point Likert scale. As such, scores were compiled on perceived teaching importance (1 = extremely unimportant, 5 = very important) and actual teaching performance (1 = very poor, 5 = very good). Then, the assessment of teaching needs was calculated by subtracting the performance scores from the importance scores (< 0 = minimum need, 0.1–1 = partial need, and > 1 = maximum need). The Cronbach’s alpha for internal consistency and reliability was 0.956 (dimensional values ranging from 0.825 to 0.925) in this study.

### Data collection and processing

Data were collected using electronic and paper forms. We distributed electronic questionnaires through an open online platform, Questionnaire Star. First, collectors were hired at various data collection points and given one-to-one online training by this study’s researchers. Training contents included the study purpose and significance, questionnaire completion requirements, methods of data collection, interpretation of scale contents, and matters needing attention during data collection. Trained data collectors explained the study purpose and significance to participants prior to investigation. After obtaining informed consent, the type of questionnaire (i.e., electronic or paper) was selected based on individual willingness. All questionnaires were completed anonymously and collected on-site. The address of the electronic questionnaire for the account teacher version is https://www.wjx.cn/jq/11354335.aspx. The address of the electronic questionnaire for the student version is https://www.wjx.cn/m/11376903.aspx.

### Statistical methods

Data were analyzed using the IBM SPSS 22.0 statistical software (IBM Corp., Armonk, NY). Descriptive statistics, including means, standard deviations, proportions, and frequencies were calculated for each study variable. A paired t-test was performed to identify discrepancies between perceived teaching importance and actual teaching performance. The rank sum test was used to compare the teaching needs of community practice teachers with different characteristics. Finally, an ordinal logistic regression analysis was used to analyze the influencing factors of teaching needs (test level was set to 0.05).

## Results

### Basic participant characteristics

In respect of the teachers paper-based questionnaire we distributed 104 questionnaires and recovered 100, of which 94 were valid questionnaires. With regard to the e-questionnaires we recovered 129 questionnaires, of which 126 were valid. This gave us an effective recovery rate of 96.1%. In respect of the students’ paper-based questionnaire we distributed 525 questionnaires and recovered 519 of which 513 were valid. With regard to the e-questionnaires we recovered 191 questionnaires of which 182 were valid. Our effective recovery rate was thus 97.9%. A total of 915 questionnaires were obtained. Of these, 220 were community practice teachers. Table 1 lists their demographic information and teaching-related factors. As shown, all teachers were female (minimum age of 20 years, 42.7% with bachelor’s degrees). The 695 remaining responses were from students, including 578 (83.2%) females.

**Table 1:** Participant characteristics (N = 220)

Variables	Numbers (N)	Percent (%)
**Community settings**		
Capital city	128	58.2
Non-capital city	92	41.8
**Age (years)**		
20–24	15	6.8
25–34	92	41.8
35–44	68	30.9
≥45	45	20.5
**Education**		
Secondary/Vocational	38	17.3
Junior college/Higher vocational colleges	88	40.0
Undergraduate	94	42.7
**Professional title**		
Junior title	93	42.3
Intermediate title	73	33.2
Senior title	54	24.5
**Teaching years**		
< 3 years	70	31.8
3–5 years	76	34.5
6–10 years	45	20.5
> 10 years	29	13.2
**Self-evaluation of teaching ability**		
Extremely excellent	32	14.5
Excellent	72	32.7
Ordinary	96	43.7
Poor	20	9.1
**Self-evaluation of training needs**		
Maximum need	47	21.3
Secondary need	143	65.0
General need	23	10.5
Minimum need	7	3.2

### Discrepancies between perceived teaching importance and actual teaching performance

Respectively, the teachers and students produced overall self-evaluated teaching needs scores of 0.61 and 0.62 (< 0 = minimum need, 0.1–1 = partial need, and > 1 = maximum need). From the teachers’ perspective, the most important need was to develop teaching skills, followed by the need to enrich teaching contents. From the students’ perspective, the most important need was to expand and enrich teaching contents, followed by developing teaching skills (Table 2).

**Table 2:** The discrepancies between perceived teaching importance and actual teaching performance

Item	Importance	Performance	Teaching needs^11,23^ (Importance -Performance)	t	P
**Teachers**					
Total score	4.26 ± 0.42	3.65 ± 0.52	0.61	18.092	<0.001
Teaching skill	4.36 ± 0.44	3.71 ± 0.59	0.65	16.874	<0.001
Teaching environment	4.30 ± 0.49^a^	3.74 ± 0.62	0.56	14.723	<0.001
Teaching content	4.17 ± 0.46^ab^	3.56 ± 0.62^ab^	0.61	18.166	<0.001
**Students**					<0.001
Total score	4.21 ± 0.52	3.59 ± 0.70	0.62	24.182	<0.001
Teaching skill	4.24 ± 0.54	3.59 ± 0.72	0.65	25.213	<0.001
Teaching environment	4.20 ± 0.55^a^	3.67 ± 0.73^a^	0.53	21.286	<0.001
Teaching content	4.23 ± 0.52^b^	3.54 ± 0.76^ab^	0.69	24.594	<0.001

Note: ^a^ representation compared with teaching skills, P < 0.01; ^b^ representation compared with use of teaching environment, P < 0.01; Perceived teaching importance is abbreviated to importance; Actual teaching performance is abbreviated to performance; The method for calculating teaching need is detailed in References 11 and 23: assessments of teaching needs were produced by subtracting the performance scores from the importance scores (< 0 = minimum need, 0.1–1 = partial need, and > 1 = maximum need)

### Univariate analysis of teaching needs

The teachers were grouped according to community setting, age, education, professional title, teaching years, self-evaluation of teaching ability, and self-evaluation of training needs. Due to the small number of secondary/vocational and junior college/higher vocational colleges, these attributes were merged into a low-educated group. Table 3 lists the results.

**Table 3:** Single factor analysis of teaching needs

Teachers (n = 220)	Numbers	Least needed (%)	Partially needed (%)	Greatest needed (%)	Z/H
**Community setting**					-2.147*
Capital city	128	31(24.2)	63 (49.2)	34(26.6)	
Non-capital city	92	24(26.1)	36 (39.1)	32(34.8)	
**Education**					2.648*
Secondary/Vocational	38	8(21.1)	17(44.7)	13(34.2)	
Junior college/Higher vocational colleges	88	15(17.0)	53(60.3)	20(22.7)	
Undergraduate	94	14(14.9)	57(60.6)	23(24.5)	
**Professional title**					12.462**
Junior title	93	23(25.2)	53(56.5)	17(18.3)	
Intermediate title	73	15(21.1)	38(51.5)	20(27.4)	
Senior title	54	7(13.8)	28(51.9)	19(35.2)	
**Teaching years**					34.762***
< 3 years	70	23(32.8)	34 (48.6)	13(18.6)	
3–5 years	76	8(10.5)	41 (53.9)	27(35.6)	
6–10 years	45	4(8.9)	21(46.7)	20(44.4)	
> 10 years	29	5(17.2)	20(69.0)	4(13.8)	
**Self-evaluation of teaching ability**					78.921***
Extremely excellent	32	13(40.6)	16(50.0)	3(9.4)	
Excellent	72	19(26.4)	34(47.2)	19(26.4)	
Ordinary	96	17(17.7)	39(40.6)	40(41.7)	
Poor	20	3(15.0)	7(35.0)	10(50.0)	
**Self-evaluation of teaching outcome**					62.737***
Extremely excellent	40	17(42.5)	18(45.0)	5(12.5)	
Excellent	76	23(30.3)	38(50.0)	15(19.7)	
Ordinary	81	15(18.5)	35(43.2)	31(38.3)	
Poor	23	2( 8.7)	9(39.1)	12(52.2)	

Only statistically significant variables were shown. *P < 0.05, **P < 0.01, ***P < 0.001

### Multifactor analysis of teaching needs

Taking teaching needs as the dependent variable (1 = minimum needs, 2 = partial needs, 3 = maximum needs), an ordinal regression analysis was conducted on six variables, including community setting, education, professional title, teaching years, self-evaluation of teaching ability, and self-evaluation of teaching outcomes. The parallel line test showed that Χ^2^ = 35.523 (p = 0.586), indicating that the slope of the model had no significant difference in the different categories of the explained variables. The ordinal logistic regression analysis could be carried out. The likelihood ratio test showed Χ^2^ = 182.158 (p < 0.001), indicating that the regression analysis had statistical significance. The goodness-of-fit test showed that deviance was 362.395 (p = 1.000), while the pseudo-determination coefficient was 0.489 for Cox and Snell, 0.542 for Nagelkerke, and 0.312 for McFadden, indicating that the model had a good fit. The results showed that the main influencing factors were community setting, education, teaching years, self-evaluation of teaching ability, and self-evaluation of teaching outcomes (p < 0.05).

Compared with community practice teachers from non-capital cities, those from the capital city had a lower degree of teaching needs (OR = 0.641, 95% CI: 0.461–0.902). The teaching needs of low-educated teachers were 15.352 times higher than those of high-educated teachers (95% CI: 1.253–26.815). Teachers with < 3 years of teaching experience had higher teaching needs (OR = 3.280, 95% CI: 1.153–10.075) than those with > 10 years of experience. Compared with teachers who self-evaluated their teaching outcomes as poor, those who reported extremely excellent (OR = 0.362, 95% CI: 0.220–0.701), excellent (OR = 0.638, 95% CI: 0.426–1.102), and ordinary (OR = 0.714, 95% CI: 0.508–1.034) teaching outcomes had lower teaching needs. Compared with teachers who self-evaluated their teaching abilities as poor, those who reported extremely excellent (OR = 0.536, 95% CI: 0.313–0.934), excellent (OR = 0.805, 95% CI: 0.518–1.269), and ordinary (OR = 0.737, 95% CI: 0.413–1.322) had lower teaching needs. Table 4 lists the results.

**Table 4:** Multifactor analysis of teaching needs

Variables	β	SE	Wald χ2	P	OR	95%CI
**Community setting**(Take non-capital city as a reference)						
Capital city	-0.440	0.158	6.871	0.006	0.641	0.461–0.902
**Education**(Take higher education as a reference)						
Lower education	2.516	1.163	4.572	0.023	15.352	1.253–26.815
**Teaching years** (Take > 10 years as a reference)						
< 3 years	1.202	0.612	4.372	0.031	3.280	1.153–10.075
**Self-evaluation of teaching outcome**(Take poor as a reference)						
Extremely excellent	-0.852	0.282	10.082	0.001	0.362	0.220–0.701
Excellent	-0.425	0.218	3.795	0. 032	0.638	0.426–1.102
Ordinary	-0.359	0.148	3.144	0.028	0.714	0.508–1.034
**Self-evaluation of teaching ability**(Take poor as a reference)						
Extremely excellent	-0.636	0.289	5.017	0.015	0.536	0.313–0.934
Excellent	-0.216	0.202	3.812	0.040	0.805	0.518–1.269
Ordinary	-0.112	0.188	0.239	0.038	0.737	0.413–1.322

Only statistically significant variables were shown

## Discussion

By gathering data on the perspectives of teachers and students, this study objectively evaluated teaching needs based on two critical elements, including perceived teaching importance and actual teaching performance. Regardless of the evaluation perspective, several needs were obvious. When teaching needs were solely evaluated based on the factor of cognition (perceived teaching importance), all behaviors were considered essential aspects of teaching activities. This emphasizes the equal nature of their importance when formulating teacher training plans. Here, the classification of “important” may originate from theoretical knowledge, teaching experience, or personal preferences [23]. To improve teaching outcomes, it is effective to divide training areas into focused and less-focused aspects through targeted teaching training interventions [24, 25]. However, it is easy to ignore important teaching behaviors if current performance is the only consideration. In this regard, the gap between perceived teaching importance and actual teaching performance should quickly be identified and placed into the most-focused area. Further, more attention should be paid to areas that are considered very important, but which are not currently performing.

When evaluating teaching needs solely from the teacher perspective, a needs assessment is also inaccurate. For example, teachers believed the largest gap was “teaching skills,” while students thought the largest gap was “teaching contents.” In teaching activities, teachers usually spontaneously improve their teaching skills through various methods, with substantial time contemplating how to improve their teaching abilities and participating in training programs [26]. Undoubtedly, teaching skill is very important. It is the core and essence of the professional teaching ability. It can help teachers achieve their teaching goals and organize the teaching process both scientifically and reasonably [27, 28]. Teaching skill is also an indispensable component of training programs [29]. From the student perspective, there are greater concerns about the diversification of teaching contents, including rich materials that arouse sufficient learning enthusiasm. As the “learner-centered” approach emphasizes the dominant position of the student, their motivation and learning enthusiasm must be considered in conjunction with the teacher’s accurate grasp of the teaching contents [30, 31]. However, teaching contents in China are uniformly developed by the Ministry of Education, which rarely considers student learning interest. Our results suggest that it is equally important to select and implement teaching contents that students find interesting.

This study also found that teachers from the capital city had lower teaching needs. This is likely because economic development is connected with educational development. Indeed, the capital city features a robust economy, abundant educational resources, and can attract high-quality teachers. Teaching levels are also relatively high, which better meets learning needs [32]. Compared to teachers with higher education, those with lower education had higher teaching needs. Here, lower education entails limited learning and teaching experience, which makes it difficult to use multiple teaching skills. In turn, it is harder to achieve teaching objectives and effectively use resources in the teaching environment. When attempting to grasp teaching contents, the learning effect is also influenced when most of the syllabus pertains to rote information. Ni and Liu [33] reported that nursing undergraduates did not reach a satisfactory level when considered learning effect. In China, the backward development of community health services and imperfect construction of community practice teaching has hindered the establishment of systematic standards for admitting and evaluating community practice teachers. This has resulted in uneven levels of teacher education, such that only 12.5% of community nurses in China have bachelor’s degrees or above [34]. In other words, practical teachers do not typically have high education levels, which emphasizes the urgency of strengthening training provisions, especially for low-educated teachers.

Compared to teachers with > 10 years of teaching experience, those with < 3 years had higher teaching needs. This indicates that sufficient teaching experience is conducive to improved teaching competence. In this study, 67.3% of teachers had been practicing < 5 years, suggesting general youth and inexperience. Most European countries divide training for community practice teachers into discrete stages, including novice, preliminary competence, competence, mastery, and authority. In this arrangement, teachers are only qualified for independent practice upon reaching high competency; indeed, community practice teachers typically have more than 10 years of experience [35, 36]. This suggests that substantial improvements can be made to address the teaching needs of community practice teachers in China. In particular, relevant departments should keenly focus on cultivating the abilities of community practice teachers with < 3 years of experience.

## Conclusion

This study found that the results of assessing teaching needs from the perspectives of teachers and students are different. It is important to solicit objective perspectives from teachers and students when conducting needs assessments. This study also found that teaching needs were influenced by many factors, including community setting, education, teaching years, self-evaluation of teaching ability, and self-evaluation of teaching outcomes. In this regard, departments should pay more attention to teachers with low academic qualifications, < 3 years of teaching experience, and roots in non-provincial capital cities, as these individuals will require additional efforts to strengthen relevant attributes. Furthermore, the education department should pay more attention to teacher feedback on practical outcomes and teaching abilities, as this can be used to construct the best teacher development plans.

### Limitations

This study had some limitations. First, teaching needs are dynamic. As the analyses were based on cross-sectional data, it is difficult to clearly explain dynamic changes. Second, this study used convenient sampling, which may entail the risk of selection bias. In this regard, unaccounted factors may have substantially impacted sample representativeness. Future studies should use more objective sampling methods, such as cluster sampling, random sampling, or systematic sampling.

## Data Availability

The datasets generated during and/or analyzed during the current study are available from the corresponding author on reasonable request.
